# Combining self-organizing maps and biplot analysis to preselect maize phenotypic components based on UAV high-throughput phenotyping platform

**DOI:** 10.1186/s13007-019-0444-6

**Published:** 2019-05-28

**Authors:** Liang Han, Guijun Yang, Huayang Dai, Hao Yang, Bo Xu, Heli Li, Huiling Long, Zhenhai Li, Xiaodong Yang, Chunjiang Zhao

**Affiliations:** 1 Key Laboratory of Quantitative Remote Sensing in Agriculture of Ministry of Agriculture, Beijing Research Center for Information Technology in Agriculture, Beijing, 100097 China; 2 College of Architecture and Geomatics Engineering, Shanxi Datong University, Datong, 037003 China; 3 National Engineering Research Center for Information Technology in Agriculture, Beijing, 100097 China; 4 College of Geoscience and Surveying Engineering, China University of Mining and Technology (Beijing), Beijing, 100083 China

**Keywords:** SOM, Biplot, UAV, Maize, High-throughput phenotyping

## Abstract

**Background:**

With environmental deterioration, natural resource scarcity, and rapid population growth, mankind is facing severe global food security problems. To meet future needs, it is necessary to accelerate progress in breeding for new varieties with high yield and strong resistance. However, the traditional phenotypic screening methods have some disadvantages, such as destructive, inefficient, low-dimensional, labor-intensive and cumbersome, which seriously hinder the development of field breeding. Breeders urgently need a high-throughput technique for acquiring and evaluating phenotypic data that can efficiently screen out excellent phenotypic traits from large-scale genotype populations.

**Results:**

In the present study, we used an unmanned aerial vehicle (UAV) high-throughput phenotyping (HTP) platform to collect RGB and multispectral images for a breeding program and acquired multiple phenotypic components (or traits), such as plant height, normalized difference vegetation index, biomass accumulation, plant-height growth rate, lodging, and leaf color. By implementing self-organizing maps and principal components analysis biplots to establish phenotypic map and similarity, we proposed an UAV-assisted HTP framework for preselecting maize (*Zee mays* L.) phenotypic components (or traits).

**Conclusions:**

This framework gives breeders additional information to allow them to quickly identify and preselect plants that have genotypes conferring desirable phenotypic components out of thousands of field plots. The present study also demonstrates that remote sensing is a powerful tool with which to acquire abundant phenotypic components. By using these rich phenotypic components, breeders should be able to more effectively identify and select superior genotypes.

**Electronic supplementary material:**

The online version of this article (10.1186/s13007-019-0444-6) contains supplementary material, which is available to authorized users.

## Background

With the imminent threat of environmental deterioration, natural resource scarcity, and rapid population growth, mankind is facing an unprecedented challenge of producing sufficient food to ensure global food security in the coming decades [1, 2]. It is estimated that farmers must produce 70% more food by 2050 to feed a population expected to reach about 9.6 billion [3]. Improving genetic gains and maintaining a stable food supply are effective measures for plant breeders and geneticist to alleviate the current situation. In the last two decades, crop sequencing technology has developed rapidly, allowing the whole genome to be sequenced rapidly at low cost. However, because of the lack of assistant phenotypic knowledge, methods for rapid identification of desirable traits have advanced little [4, 5]. With increasing demand for rapid phenotyping of large numbers of lines and to accelerate progress in breeding for novel traits, phenotyping is often considered the bottleneck of crop breeding [6].

Recent advance in high-throughput phenotyping (HTP) technologies has provided a positive response to narrow the gap between the wealth of genomic data with phenotypic data [7]. HTP technologies allow large numbers of plants to be measured in a non-destructive manner with accuracy and precision. Initially, high-throughput phenotyping was applied in controlled environments, such as greenhouses and growth chambers, to collect phenotypic data from model organisms [8]. This is indoor shoot-based phenotyping that have an advantage in characterizing individual plants grown in pots, and not limited by overlapping canopies and variable environmental conditions due to soil, temperature, water etc. However, the main concern for many breeders is that the complex traits obtained by using HTP technologies in controlled environments may not be fully replicated in the field, so phenotyping in field conditions remains a bottleneck that hinders advances in breeding [5, 6, 9].

With continuous advances in proximal sensing, field-based HTP has become widespread in the breeding programs. Recently, several field-based HTP platforms were developed to measure phenotypic traits, including ground-based HTP platforms [10–12] and aerial-based HTP platforms [13–15]. Ground-based HTP platforms consisting of modified vehicles have the advantages of high resolution, flexible design, and large payload, but have limitations in the portability and scale at which they can be used [16]. Compared to ground-based HTP platforms, aerial-based HTP platforms enable the rapid evaluation of the populations consisting of thousands to tens of thousands of plots and the synchronized measurements of multiple traits in an efficient manner, overcoming some limitations associated with the ground-based HTP platforms. As one of emerging technologies in aerial-based platforms, unmanned aerial vehicles (UAV) for HTP have undergone a remarkable development in recent years and been capable of gaining advantages of their portability, operability, low cost, and high spatiotemporal resolution [13, 17, 18]. UAV-HTP based on proximal remote sensing has been envisaged to bridge the gap between ground-based measurements and satellite observations [19]. Traditional ground-based phenotyping techniques are time-consuming, labor intensive and impractical for large-scale operations [20]. Despite the advantages of satellite remote sensing in large-scale observation, it remains some limitations, such as low resolution, long revisit period and high susceptibility to water vapor [21]. In conclusion, compared with other technologies, UAV-HTP offers excellent opportunities for rapid and non-destructive extraction of crop phenotypic information in the field.

Currently, several structural and physiological agronomic traits suitable for HTP have been proposed for use in breeding programs, including but not limited to the normalized difference vegetation index (NDVI) [11, 22, 23], biomass accumulation [21, 24], plant height [25, 26], plant-height growth rate [15, 27, 28], lodging [29, 30], leaf color [24, 31], and yield [32–34]. Previous research has demonstrated that measurements provided by HTP platforms are highly correlated with manual reference measurements [26, 34, 35]. By using HTP technologies capable of collecting phenotypic data at multiple time points or throughout the season, researchers can better understand how traits develop, allowing better optimization of genotypes through selection in breeding programs [36].

A preliminary approach with easily measurable phenotypic traits provides a chance to select genotypes [37]. Cluster and correlation analyses seem to be a promising approach for identifying potential associations between phenotype and genotype [38, 39], which clarifies gene co-expression and phenotypic similarity. Self-organizing maps (SOMs) are a type of artificial neural network invented by Kohonen [40] that are trained by using unsupervised learning to project high-dimensional, complex data onto a two-dimensional grid. This reduces dimensionality and enhances the visualization of clustering [41, 42]. A principal components analysis (PCA) biplot highlights the extent to which the objects in rows (samples) differ from the objects in columns (features) [43]. In this context, a PCA biplot shows the largest patterns in the data in terms of how the phenotypic components differ in different genotypes.

In the present study, we used a UAV HTP platform to collect RGB and multispectral (spectral bands: green, red, red-edge and near-infrared) images for a breeding program and acquired multiple phenotypic components (or traits). The specific objectives for this study were (i) to propose an UAV-assisted HTP framework to establish phenotypic maps and similarities, (ii) to identify selection strategies for different breeding targets or multiple phenotypic components, and (iii) to assess the potential for using UAV field-based HTP platforms for selection decisions in a large breeding program.

## Methods

### Field trials

Field breeding trials included a natural population and a doubled-haploid population (i.e., 800 maize plots). The natural population assessed in this study consisted of 482 maize plots divided into three subpopulations based on differences in genetic background: Mixed, temperate (TEM), and tropical or subtropical (TST). Of the 482 plots, 106 were from the mixed subpopulation, 162 were from the TEM subpopulation, and 214 were from the TST subpopulation.

The field trials were conducted at the research station of Xiao Tangshan National Precision Agriculture Research Center of China, Changping District of Beijing City (115°50′17″–116°29′49″E, 40°20′18″–40°23′13″N). The study area is in a warm temperate zone with a semi-humid continental monsoon climate. The annual average temperature is 11.8 °C, and the annual average precipitation is 550.3 mm. Eight hundred plots were arranged across a field measuring approximately 30 m by 196 m, with a spacing of 0.8 m between plots. Each plot consisted of three 2.4-m-long rows, each containing approximately 27 plants spaced 25 cm apart. All plots were planted by using a seeder on May 15, 2017.

### HTP platform and data acquisition

**Fig. 1:**
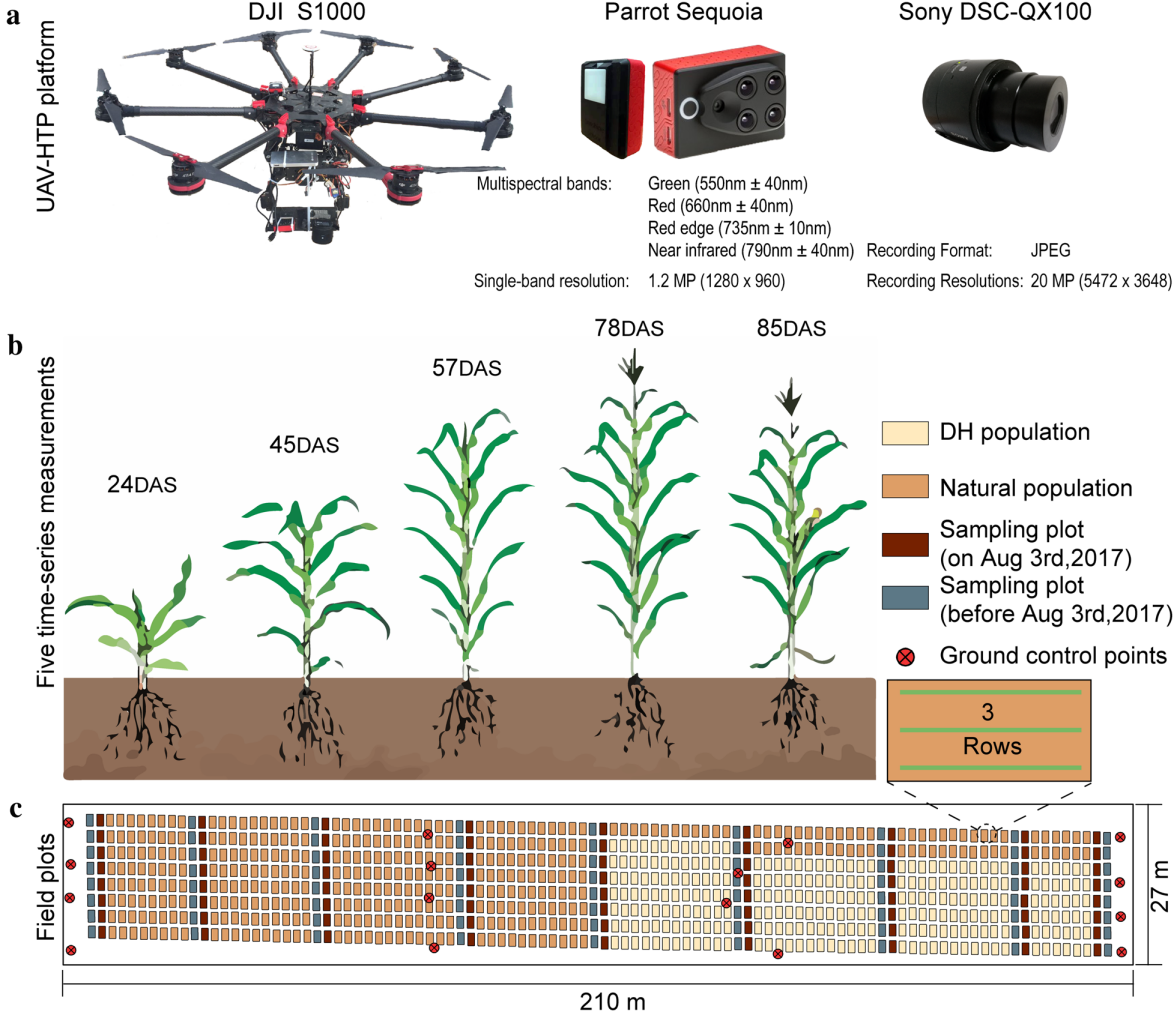
Schematic diagram of trial design and field layout

The two cameras used in this study were mounted on an UAV HTP platform (DJI Spreading Wings S1000, SZ DJI Technology Co., Shenzhen, China). The first camera was a Sony digital RGB high-resolution camera (DSC-QX100, 5472 × 3648 pixels, Sony Electronics, Inc., Tokyo, Japan) with the ISO and shutter speed set at 160 and 1/2000, respectively. The second camera was a Parrot Sequoia multispectral camera (1280 × 960 pixels, MicaSense Inc., Seattle, USA) that combines four monochrome sensors (green: 550 nm, red: 660 nm, red-edge: 735 nm, near-infrared: 790 nm) and can simultaneously capture four different band images with a 10 nm bandwidth (half maximum bandwidth) for the red-edge band and a 40 nm bandwidth for the green, red, and near-infrared bands. A sunshine sensor was used with the Sequoia sensors to minimize errors caused by variations in ambient light during acquisition (Fig. 1a).

Flight paths were designed by using the DJI ground station (SZ DJI Technology Co., Shenzhen, China) to ensure 80% forward overlap and 75% side overlap, yielding six strips. To build the digital elevation model (DEM), the above-ground-level (AGL) parameter for the first flight was set to 40 m, yielding a ground-sampling distance of 0.72 cm. The AGL parameter for four other flights was set to 60 m, yielding a ground-sampling distance of approximately 1.33 cm. The radiometric calibration images were captured on the ground before and after each flight by using a calibrated reflectance panel (MicaSense Inc., Seattle, USA). Prior to the first flight, 16 ground control point (GCP) markers were arranged evenly over the experimental site and were measured by using a differential global positioning system (DGPS, South Surveying & Mapping Instrument Co., Ltd., Guangzhou, China) with millimeter accuracy.

Images were acquired five times over the period spanning vegetative to reproductive growth (Fig. 1b). Table 1 details the flight conditions. The leaf collar method of Ritchie [44] was used for staging maize plant growth. Due to differences in genotype, heterogeneity appears in the growth and development at the plot scale. Therefore, the growth stage in Table 1 was determined by using the 50% majority rule.

**Table 1:** Flight conditions for acquiring images over period spanning vegetative to reproductive growth

Flight	Date	DAS^a^	AGL	Growth stage
1	2017-06-08	24	40	V4
2	2017-06-29	45	60	V10
3	2017-07-11	57	60	V14
4	2017-07-28	78	60	VT
5	2017-08-04	85	60	R1

^a^Days after sowing

### Plant sampling and measurements

A total of 72 plots served as sampling plots for destructive biomass measurements and plant-height measurements. Because of the consumption caused by destructive sampling, another 72 plots were selected for the measurement on August 3, 2017 (Fig. 1c). Three plants were selected at random from the middle of the sampling plots to measure plant height and fresh biomass. Plant height was manually measured by using a telescopic leveling rod. The mean height of three plants was used as plant height at the plot scale for the ground truth. The three selected plants were then used for destructive biomass sampling. The fresh biomass was sealed in plastic bags and weighed on the same day. By calculating the actual number of plants per sampling plot, the mass was rescaled to kg/m^2^. We visually determined the color of the positive leaves at the plot scale and recorded the results. From July 1 to 10, 2017, lodging occurred in some plots due to frequent strong winds and rainfall. A field investigation was done for 800 plots and the results for were recorded for root lodging, stem breaking, and stem lodging. Table 2 gives the dates for sampling and measurements.

**Table 2:** Timing of plant sampling and measurement

Plant height	Fresh biomass	Lodging	Color
2017-06-29 (45)	2017-06-29 (45)	–	2017-06-30 (46)
2017-07-11 (57)	2017-07-11 (57)	2017-07-12 (58)	–
2017-07-29 (79)	–	–	–
2017-08-03 (84)	2017-08-03 (84)	–	–

The date and days after sowing (in parentheses) are given for each task

### Image processing and data extraction

Following UAV image acquisition, RGB images and multispectral images were processed by using two different software applications. For RGB images, we used Agisoft PhotoScan (version 1.3, Agisoft LLC, St. Petersburg, Russia) to generate orthomosaic and digital surface models (DSMs) of each flight with the GCPs to optimize the camera position and ensure precise alignment [45]. A workflow (Han et al. 2018) was applied to create an area of interest for each plot by using the orthomosaics and to build the DEM by using the DSMs. Multispectral images were processed with Pix4D Mapper Pro software (version 4.0, PIX4D, Lausanne, Switzerland). Pix4D Mapper Pro has advantages in radiometric calibration and vegetation index calculation and offers some important processing steps similar to Agisoft Photoscan, such as aligning photos, importing GCPs and geographic references, building dense point clouds, and generating DSM and orthomosaics. Radiometric calibration was done by using the Pix4D Mapper Pro software with radiometric calibration images with known reflectance values provided by MicaSense. NDVI (or other vegetation indices) maps were then produced by using the index calculator.

**Fig. 2:**
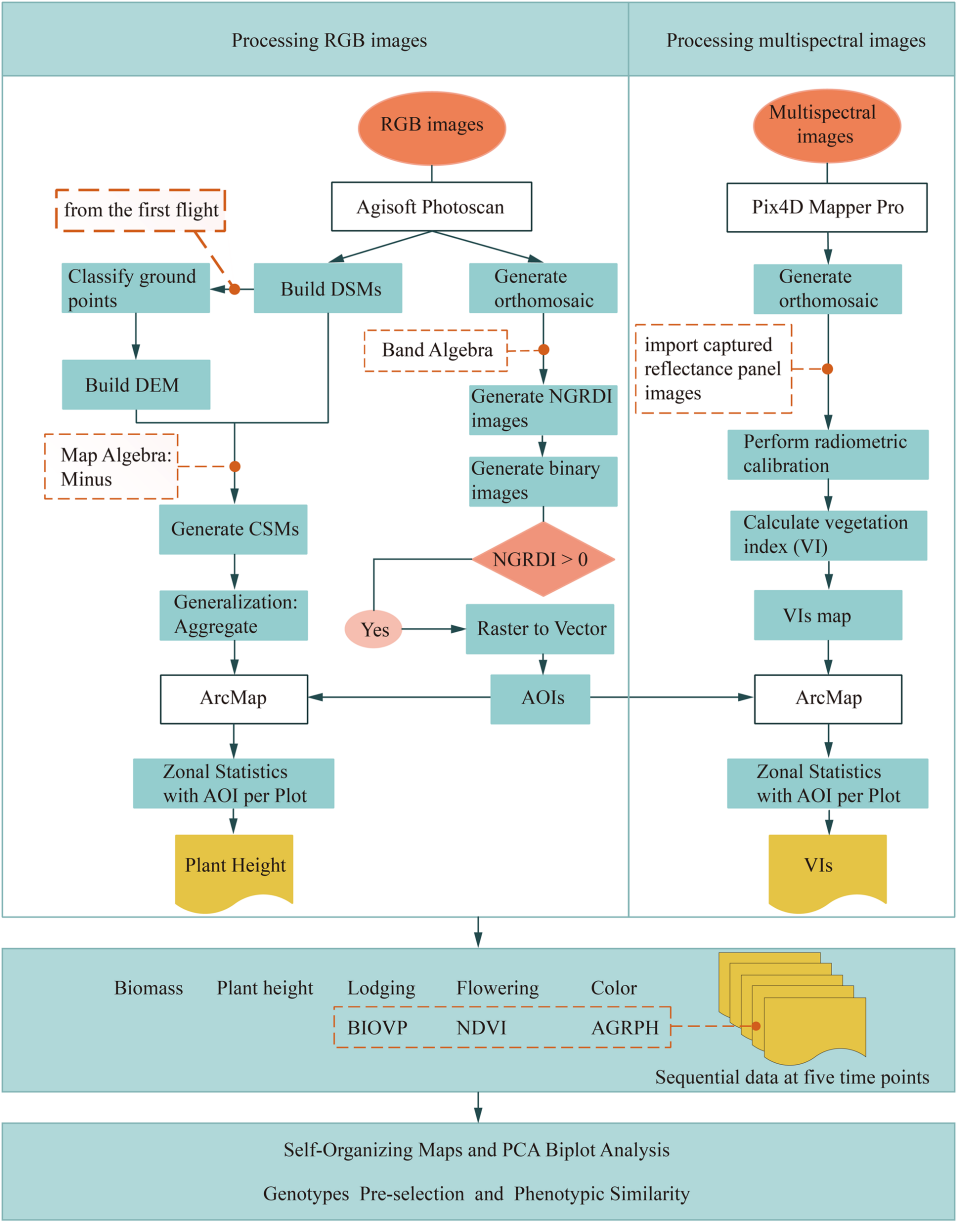
Flowchart illustrating data-acquisition and analysis methodology

Crop surface models (CSMs), which are widely used to extract crop height, were obtained by subtracting the DEM from the DSMs. By using the areas of interest, a workflow [21] was applied to extract phenotypic data such as plant height and NDVI for each plot in the field from CSM and NDVI maps. Figure 2 illustrates the complete preprocessing chain.

After transforming a RGB image into HSL (i.e., hue, saturation and lightness) color space by using ENVI software (version 4.5, Esri Inc., Redlands, USA) and combining with field-sampled data, we used the hue value in HSL color space to cluster the population and classified the population into three leaf colors. Flowering was defined as a dichotomous variable that distinguishes whether flowering occurs at the fourth time point, as judged by visual observation of an orthomosaic. Average growth rate of plant height (AGRPH) is the increment in plant height per day between two adjacent time points [15]. Fresh biomass and BIOVP (a volume metric to estimate crop biomass within certain spatial ranges) were calculated following Han [21]. The identification of maize lodging was implemented following Han [29].

### Self-organizing map and hierarchical cluster analysis

Selective breeding requires analysis of the relationships between multiple phenotypic traits and focuses on genotypes that are differentially expressed and co-expressed under the same environmental conditions. Differential expression can be accomplished using statistically significant difference test, while co-expressed genotypes require cluster analysis to examine the relationship between individuals or groups at the multiple-traits level. To explore co-expressed genotypes and identify underlying agronomic groups with similar phenotypic components, we performed two-step clustering to isolate 482 samples with nine dimensions that we standardized to values by using the mean and standard deviation. Two-step clustering was done for a pre-clustering by using the self-organizing map (SOM) method, which generates a simplified representation of the original data set and converts nonlinear statistical relationships between high-dimensional data into simple geometric relationships between points on a two-dimensional map [46]. The pre-clusters were then subjected to agglomerative hierarchical clustering (AHC), which projects similar samples onto the same neuron. Finally, AHC analysis revealed the neighboring neurons of the topological map belonging to the same final cluster. A tree diagram was used to illustrate the arrangement of the clusters produced by AHC and to understand and identify clusters.

**Fig. 3:**
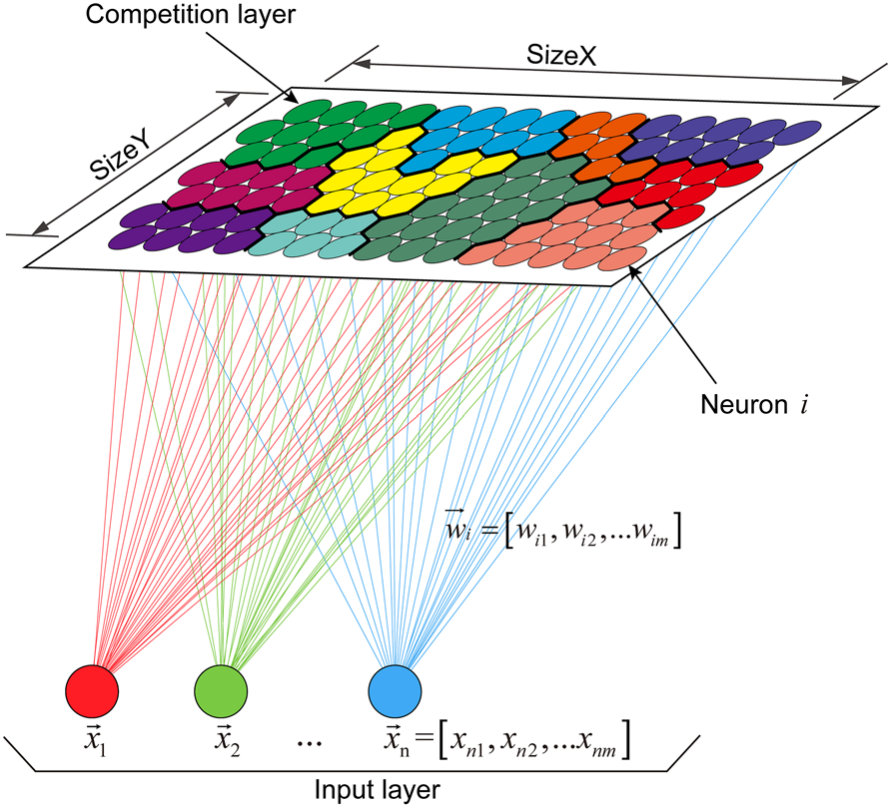
Structure of 15 × 7 two-dimensional SOM network. The schematic illustration shows how to train the SOM. The *N* input vectors in the input layer are mapped onto a two-dimensional competition layer represented by vectors containing the weights. Each neuron $i$ has a weight vector $\vec{w}_{i}$ of the same dimensionality *m* as the input vectors ($\vec{x}_{n}$). By hierarchical clustering, the color of the map encodes the input vectors with similar properties

As shown in Fig. 3, the basic structure of the SOM network consists of an input layer and a competition layer. For mixed data in the input layer, an extra layer is created for each categorical variable, so difference distance measures for each layer. We used the Euclidean distance for the numerical variables and the Tanimato distance for the categorical variables, and then computed these distances for all weight vectors. For training, each neuron was associated with a weight vector (i.e., codebook) of the same dimensionality as the input vectors (i.e., phenotypic data), and the weight vector was updated at each iteration so that topological properties in the input layer were preserved [42].

We used the *kohonen* R package (version 3.0.8) [47] to perform the two-step clustering as follows [48]:

- *Step 0* Select the size (including size X and size Y, i.e., the number of neurons), topology type (rectangular or hexagonal), and neighborhood function (Bubble or Gaussian).
- *Step 1* Each neuron is assigned a random codebook vector ($\vec{w}_{i}$) with the same dimensionality *m* as the input data ($\vec{x}_{n}$).
- *Step 2* Select a data point at random from the training data and feed it into the SOM.
- *Step 3* Find the neuron whose codebook vector is most similar to the input data. This neuron is called the best matching unit (BMU). Similarity is calculated by using the Euclidean distance as numerical variable or the Tanimato distance as categorical variable.
- *Step 4* Move the BMU closer to the data point. The distance that the BMU moves is determined by the learning rate α, which decreases after each iteration.
- *Step 5* Adjust the codebook vector in the BMU’s neighbors towards the chosen data point, depending on the neighborhood radius *r* whose value decreases after each iteration.
- *Step 6* Update the learning rate α and neighborhood radius *r*, and repeat steps 2–5 for *N* iterations until the neuron positions stabilize.
- *Step 7* Cluster the stabilized codebook vectors by using AHC with Ward’s minimum variance method linkage. The input data are separated into groups of similar properties, which are presented in different colors. Estimate the optimal number of clusters by using the *NbClust* R package (version 3.0) [49] with majority rule [50]. This provided 30 indices that determine the number of clusters in a data set, but not all of the indices always work with all distance matrices, especially for a mix of numerical and categorical data. Therefore, only 20 applicable indices were used in the end (Additional file 1).

Finally, we ran SOM with the following parameters according to the guidelines for building a SOM reported by Das [51] and Wendel [52] : SOM size: 15 × 7; 5000 iterations; learning rate: 0.05, hexagonal topology and Gaussian neighborhood function. We resolved the clusters derived from the SOM map into a set of clustering rules by using the *rpart* [53] (version 4.1-15) and *rpart.plot* [54] (version 3.0.7) R package and evaluated clustering quality. To facilitate interpretation of the clusters, we used the *ComplexHeatmap* R package (version 1.99.5) [55] to enhance the visualization of the clustering results by making a heat map with a dendrogram. The *UpSetR* R package (version 1.3.3) [56] used to visualize the set intersections was also used to identify clusters with typical phenotypic-component patterns.

Wilcoxon rank-sum test was used to compare each cluster mean with the total population (without clustering) mean, and observe whether a phenotypic component was overexpressed (above the total population mean) or underexpressed (below the total population mean) in different clusters.

### Analysis of principle components analysis bioplot

Two-step clustering was followed by biplot analysis associated with principle components analysis (PCA). By using the *FactoMineR* [57] and *factoextra* [58] R packages, the biplot was analyzed with a new dataset based on two-step clustering to characterize the relationship between phenotypic components and to identify the leading components. Biplots based on simple bivariate scatter plots can show inter-unit distances and indicate clustering of units in addition to displaying variances in and correlations between the variables [59]. The new dataset was projected onto two dimensions to approximately preserve the distances between the samples. The points in the biplot approximate the row (sample) information and the vector approximates the column (i.e., phenotypic component) information. The distance between points reflects the difference between the corresponding samples. A greater distance between two points reflects a greater difference between the corresponding samples, and vice versa. The length of the arrows represents how well the phenotypic component explains the distribution of the data, whereas the angles between the arrows approximate their correlations. Therefore, when two vectors are approximately perpendicular, the correlation between the two variables is very weak, and they are essentially independent of each other. But if they are nearly parallel (antiparallel), the variables have a high positive (negative) correlation.

## Results

### Phenotypic components from high-throughput phenotyping images

**Fig. 4:**
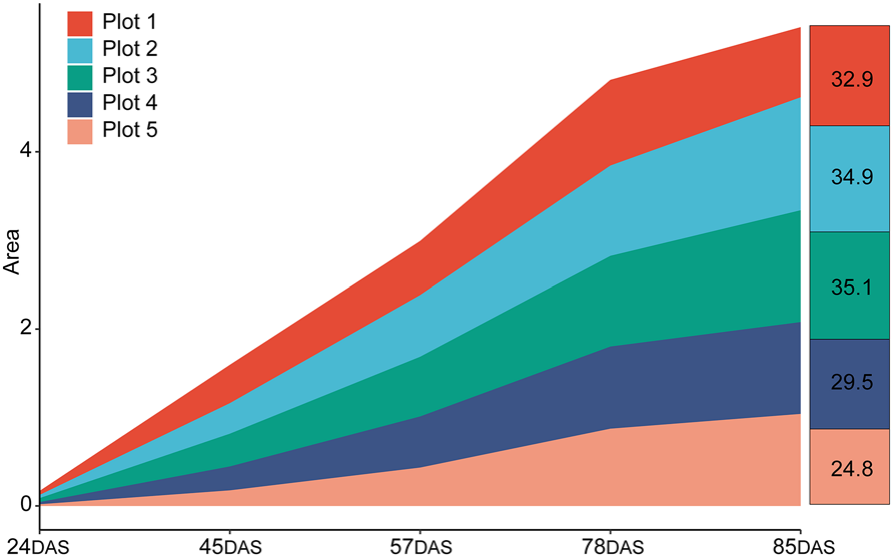
Converting time series data (BIOVP) into a numerical value by calculating the area under the polyline. The first five plots were selected as examples. The number in the right column indicated the corresponding area after conversion

The phenotypic components evaluated in this study included plant height, fresh biomass, flowering, lodging, leaf color, genetic background, NDVI, AGRPH and BIOVP. Except for genetic background, these phenotypic components were acquired by processing and analyzing digital or multispectral images from HTP. Manual grading based on field investigations led to extreme imbalance of the lodging samples, i.e., more than 80% was root lodging [29]. Therefore, we simply divided the population into two categories (i.e., lodging and non-lodging). Note that NDVI, BIOVP, and AGRPH were all time series data. For convenience, we converted the time series data into a numerical value by calculating the area under the polyline (Fig. 4). Phenotypic components were classified into three categories based on data types: numerical, dichotomous, and polytomous. Table 3 summarizes the phenotypic data evaluated in this study.

**Table 3:** Phenotypic components evaluated in this study

Number	Variable	Sensor	Data type	Explanations
1	finPH	RGB	Numerical	Plant height at fifth observation time point
2	finBiomass	Both	Numerical	Fresh biomass at fifth observation time point
3	IS_flowering	RGB	Dichotomous	Y means that the tassel has flowered, whereas N means that there is no tasseling or tassel has not flowered
4	IS_lodging	Both	Dichotomous	Y means lodging; N means no lodging
5	tColor	RGB	Polytomous	Three colors: green, green-yellow, and dark green
6	tGBK	–	Polytomous	Three genetic backgrounds: Mixed, temperate (TEM) and tropical-subtropical (TST)
7	dyNDVI	Multispectral	Numerical	Converted from NDVI data across four time points
8	dyBIOVP	RGB	Numerical	Converted from BIOVP data across five time points
9	dyAGRPH	RGB	Numerical	Converted from AGRPH data across five time points

### Clustering genotypes based on phenotypic components

**Fig. 5:**
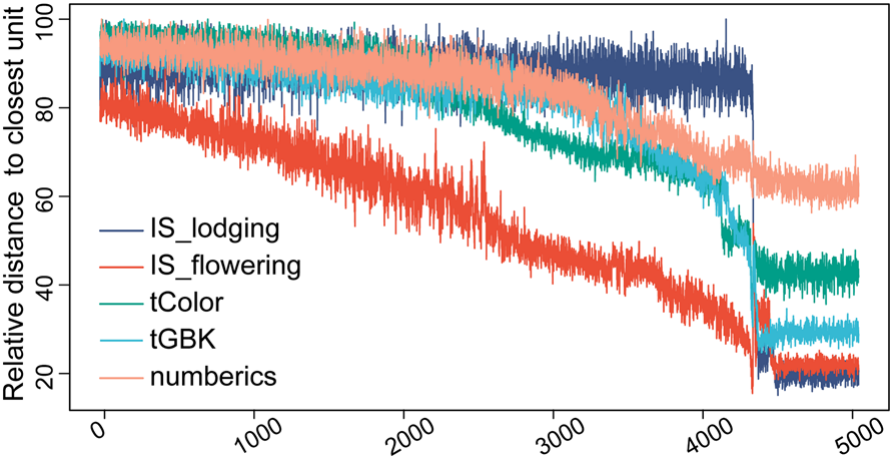
Progress of SOM training iterations for 482 samples

**Fig. 6:**
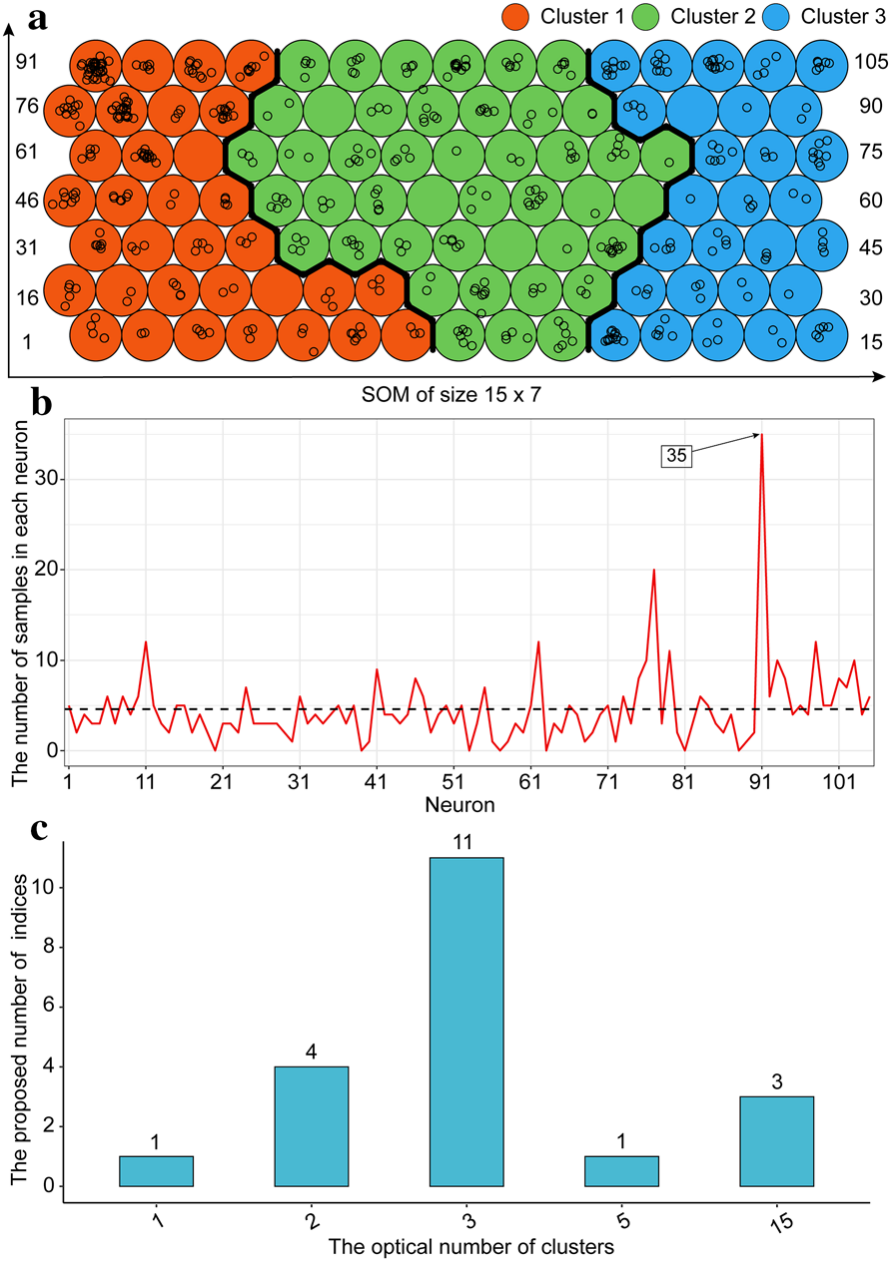
Frequency distribution of samples in SOM grid and optical number of clusters. **a** SOM grid. The numbers on the left and right were the sequence numbers of 105 neurons. **b** Number of samples in each neuron. Seven neurons were dead neurons (i.e., neurons that never won the competition for samples). **c** Determining the optical number of clusters using the majority rule and 20 indices

**Fig. 7:**
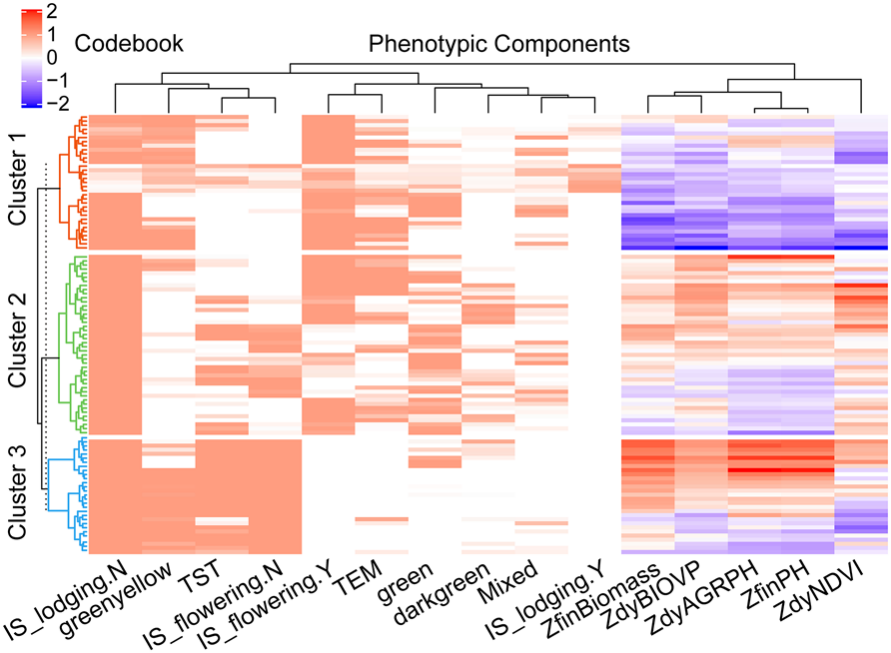
Hierarchical agglomerative clustering for codebook. Clustering the codebooks resulted in the identification of three major clusters marked in three colors, to which different genotype samples had been assigned (left: clustering samples by rows). The dendrogram on the top showed the measure of phenotypic components similarity (clustering phenotypic components by columns). Converting categorical variables into dummy variables expanded to 15 the number of phenotypic components used for clustering. The initial letter “Z” indicated that this variable was standardized

There were five input data layers. The numerics layer consisted of five continuous numerical variables (i.e., *dyAGRPH*, *dyNDVI*, *dyBIOVP*, *finBiomass* and *finPH*, see Table 3). Figure 5 shows that the relative distance to the closest unit approximately stabilizes after 5000 iterations, which means that the algorithm has converged. A total of 105 neurons in the SOM were arranged in a grid of 15 rows by 7 columns, and 482 samples were unevenly distributed among these neurons (Fig. 6a). Samples with similar phenotypic-component patterns tended to be at nearby grid locations. The 91st neuron contained 35 samples, which was the largest number of samples for a single neuron. Seven neurons, called “dead” neurons, never won the competition for samples; these accounted for less than 7% (Fig. 6b). Figure 6c shows that, based on the majority rule, 11 of the 20 indices propose three as the optimal number of clusters. In other words, these phenotypic components contributed strongly to discrimination of genotypes into three clusters. Therefore, hierarchical agglomerative clustering of 105 codebooks resulted in the identification of three major clusters, with different genotype samples assigned to these clusters (Fig. 7). After clustering, genotypes with similar phenotypic-component characteristics were grouped in the same cluster. The next section discussed co-expressed genotypes and how to identify plant phenotypic similarity.

### Phenotypic map and similarity

**Fig. 8:**
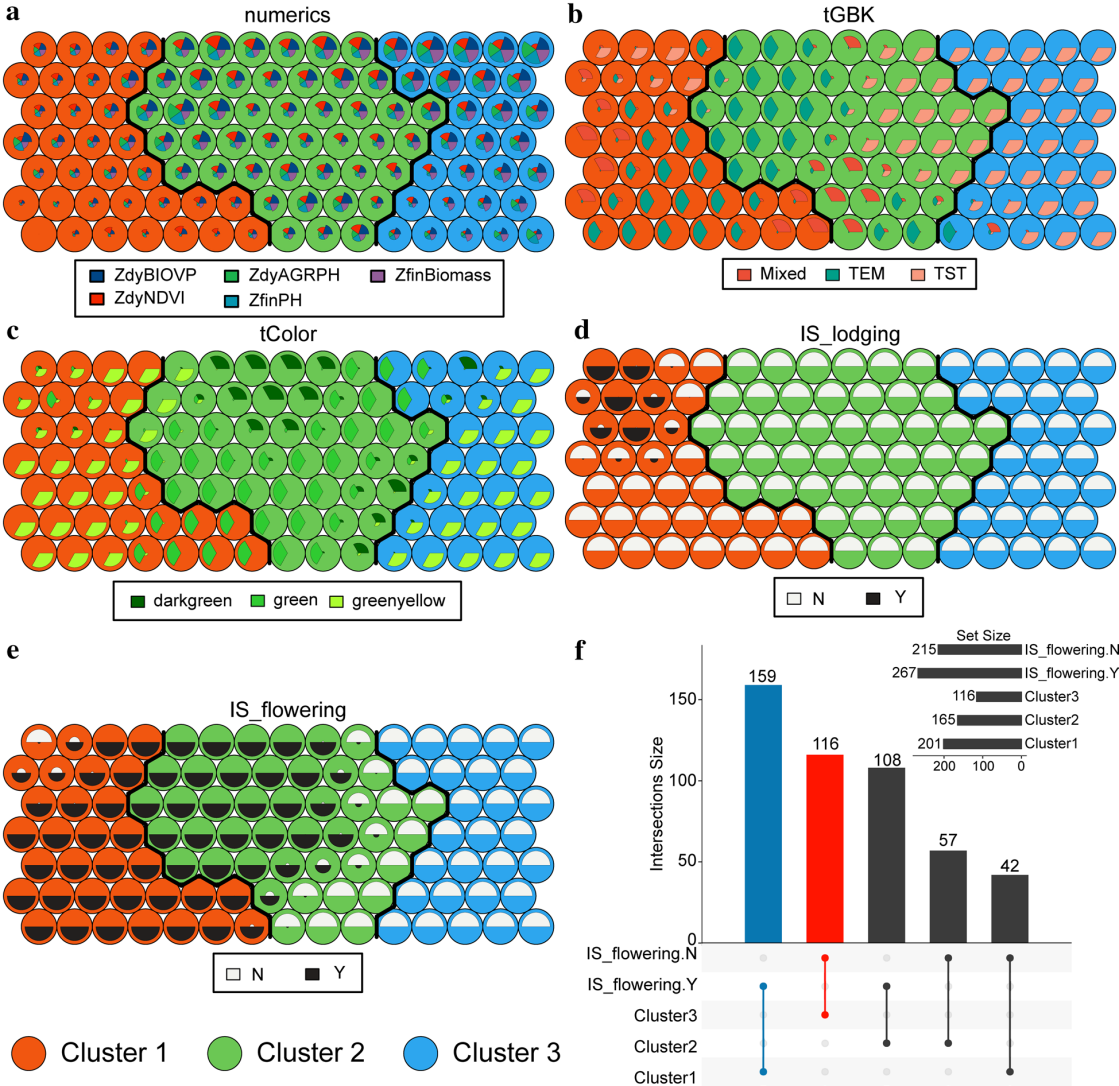
Exploring the distribution patterns of sample and phenotypic components in clusters. **a**–**e** The weight vector was visualized as a fan diagram to indicate the weight of each phenotypic component in a neuron. **f** Taking *IS_flowering* as an example, the phenotypic component patterns within clusters were identified to reveal the effect of co-expressed genotypes. The intersection of cluster 3 and the *IS_flowering*.N set gave 116 samples, which indicated that none of the samples in Cluster 3 were tasseling and flowering, accounting for 100% of the samples. This was shown with a striking color to distinguish between unrecognized patterns. The initial letter “Z” indicated that this variable was standardized

A phenotypic map was constructed by using SOM clustering visualization technology and dendrograms. This map provides important information regarding plant phenotypic similarity or dissimilarity and supports further evaluation of the phenotypic components [28]. By visualizing the weight vector on the map, we explored the patterns in which the samples and phenotypic components distributed across the three clusters. The weight vector was visualized as a “fan diagram” to indicate the weight of each phenotypic component in a neuron (see Figs. 8a–e). Closer inspection of these diagrams shows that (i) the weights of the numerical phenotypic components are relatively small in cluster 1 (Fig. 8a); (ii) the TST subpopulation dominates cluster 3, accounting for more than 85% (Fig. 8b); (iii) cluster 2 has almost no green-yellow samples (less than 5% of the samples), although they accounted for over 75% of the samples in cluster 3 (Fig. 8c); (iv) all samples containing lodging phenotypic components are in cluster 1 (Fig. 8d); and (v) none of the samples in cluster 3 are tasseling and flowering (Fig. 8e).

**Fig. 9:**
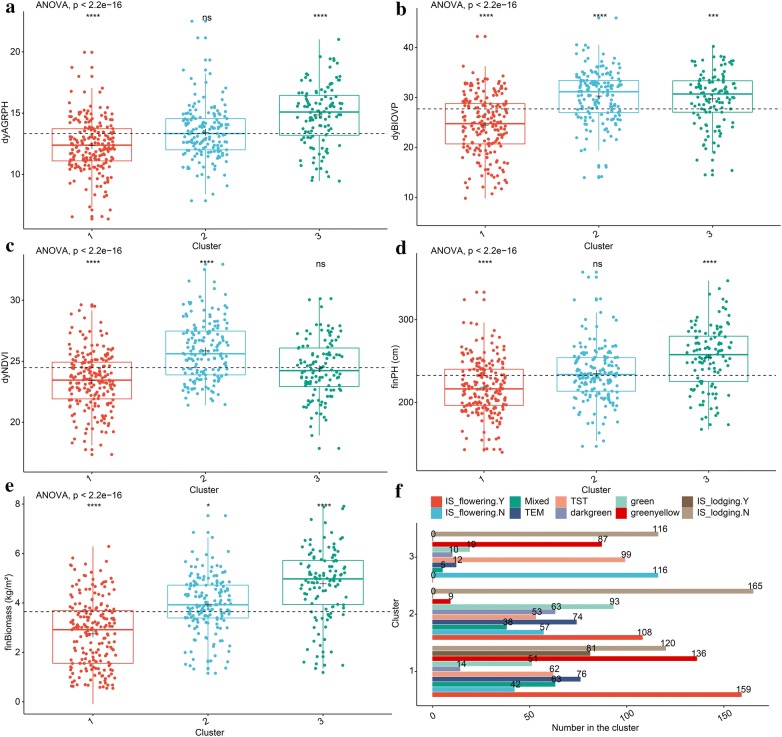
Phenotypic components differences in the three genotype clusters. **a**–**e** Five numerical phenotypic components. **f** Categorical phenotypic components. The dashed black line indicates the mean of a phenotypic component from all samples without clustering. The black plus sign indicated the mean of a given phenotypic component from each cluster. An ANOVA was used to determine whether differences exist between three-cluster means. The Wilcoxon rank-sum test was used to compare each cluster against all genotypes without clustering. The following convention for symbols indicated statistical significance: *p* > 0.05 (ns), *p* ≤ 0.05 (*), *p* ≤ 0.01 (**), *p* ≤ 0.001 (***), *p* ≤ 0.0001 (****)

As shown in Fig. 9, the overall analysis of variance (ANOVA) gives a *p* value less than 0.05, so we further compared the differences in the mean phenotypic components between each cluster and that of all genotypes without clustering. When the Wilcoxon rank-sum test was significant, the numerical phenotypic components in cluster 1 were significantly low compared with all components (i.e., without clustering), and other numerical phenotypic components except *dyNDVI* in Cluster 3 were significantly higher compared to all components. However, no significant difference appeared in the parameters *finPH* and *dyNDVI* between cluster 2 and all components. Phenotypic components with similar characteristics were considered to be co-expressed by different genotypes. Together, the visualization of the results of the analysis provided important insights into the clusters and their co-expressed patterns based on two-step clustering (Table 4).

**Table 4:** Summary of clusters and their co-expressed patterns based on two-step clustering

Cluster	Neuron size	Sample size	Description
1	33	201	Lower numerical phenotypic components, e.g., less fresh biomass and lower average growth rate of plant height
2	44	165	Green or dark green leaves, almost no green-yellow leaves
			Larger NDVI
3	28	116	Higher numerical phenotypic components, e.g., more fresh biomass and plant height
			Longer vegetative growth stage

**Fig. 10:**
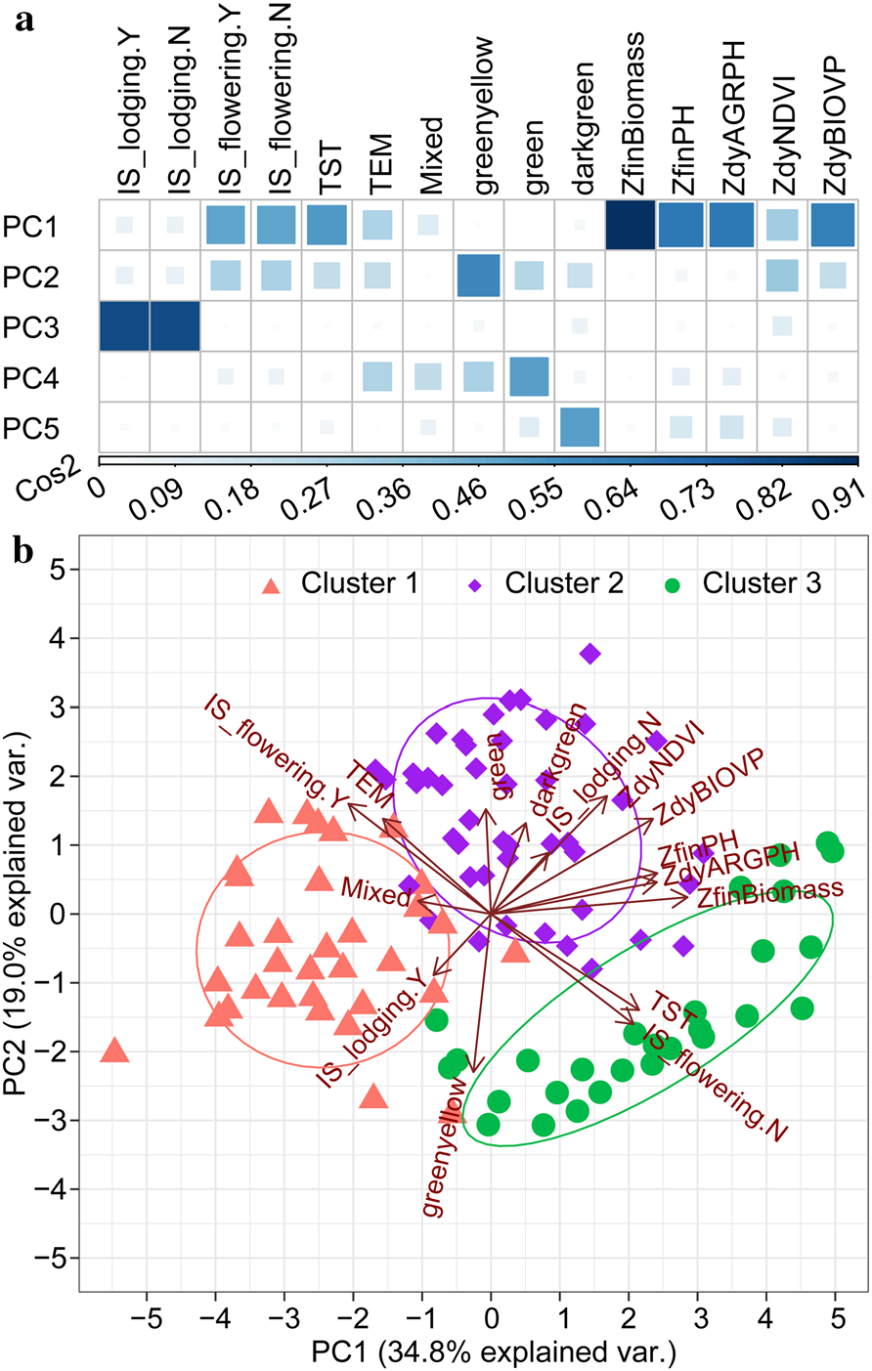
PCA biplot showing phenotypic similarity and relationship between genotypes and phenotypic components. **a** Visualization of quality of representation of variables in top five dimensions. *Cos2* is the quality of representation of the variables in the principal component maps. **b** Biplot analysis for phenotypic similarity. Correlated phenotypic components and genotype samples were located in the same quadrant. The cosine of the angle between vectors indicated correlation between phenotypic components. Highly correlated phenotypic components pointed in roughly the same direction. Nearby points in the biplot represented samples with similar patterns; these were colored according to clustering. The initial letter “Z” indicated that this variable is standardized

Turning now to phenotypic similarity, Fig. 7 provides an overview, via a dendrogram, of the measurement of the similarity between phenotypic components by using HAC with the spearman correlation distance. The top five principal components (PCs) explained 86.2% of the variation. A high *Cos2* (square cosine, squared coordinates) indicates that the phenotypic component is well represented in the principal component [58]. Therefore, *ZfinBiomass* (i.e., standardized fresh biomass) was more important to interpret PC 1, and *IS_lodging* was more important to interpret PC 3 (Fig. 10a). Figure 10b presents a biplot that simultaneously plots information on genotype samples and phenotypic components, further revealing the phenotypic similarity and the relationship between genotypes and phenotypic components. PC 1 explained 34.8% of the variation and PC 2 explained 19.0% of the variation. A high positive correlation existed between the numerical phenotypic components, which were negatively correlated with *IS_lodging.Y*. Lodging had a negative impact on crop biomass, plant height, NDVI, and so on. A significant positive correlation occurred between *TST* and *IS_flowering.N* and between *TEM* and *IS_flowering.N*, which we attributed to the fact that the experimental site was located in a temperate zone, so the *TEM* subpopulation was tasseling and flowering in the V1 stage, whereas the *TST* subpopulation required more accumulated temperature from vegetative overgrowth to reproductive growth. The correlation between *greenyellow* and *IS_lodging.Y* was interesting because it seemed to indicate that the green-yellow leaf samples were more prone to lodging. Similarly, a significant positive correlation occurred between *darkgreen* and *ZdyNDVI*. Interestingly, this correlation was related to the canopy pigment content, which was remotely estimated based on the NDVI.

## Discussion

### Phenotypic temporal profile

Prior studies noted the importance of phenotypic temporal profiles, providing fresh insights into the dynamic changes in phenotypic traits [15, 60] and genetic differences at various stages of plant development [27, 61]. Although UAV-HTP allows researchers to efficiently and conveniently acquire multi-temporal phenotypic data by remote sensing, integrating time-series data with other types of phenotypic data, such as leaf color, to facilitate subsequent quantitative genetic analysis remains a difficult problem. In this study, the area under the polyline is highly correlated with other phenotypic components after conversion to a single value (Figs. 3 and 10). Although this conversion preserves the multi-temporal information of phenotypic components to some extent, obvious deficiencies remain because, given equal areas, the shape of the polylines may differ. This leaves the way open for further improvements: one option would be to add some descriptive parameters such as inflection points and trends. The challenges of collecting phenotypic temporal profiles are further compounded by their observed values, which partially depend on environmental conditions and may change dramatically within a given day or between days of a given year [36].

### Phenotypic preselection

The results of this study show that two-step clustering allows genotypes to be segregated according to different phenotypic component patterns. For example, cluster 3 exhibits a pattern that is a higher numerical phenotypic components, so when we preliminarily screen genotypes for higher biomass or higher plant height, which are both key breeding targets for crop improvement, we can narrow the range of candidate populations and select genotypes only in this cluster. Grain yield is also one of the most important breeding targets for breeders. Unfortunately, the grain-yield data for this trial were largely missing and unreliable due to waterlogging caused by heavy rainfall during the harvest period. These data were therefore replaced by the historical grain-yield data. In this way, we try to further explain the rationality of clustering results produced by our pre-selection framework.

**Fig. 11:**
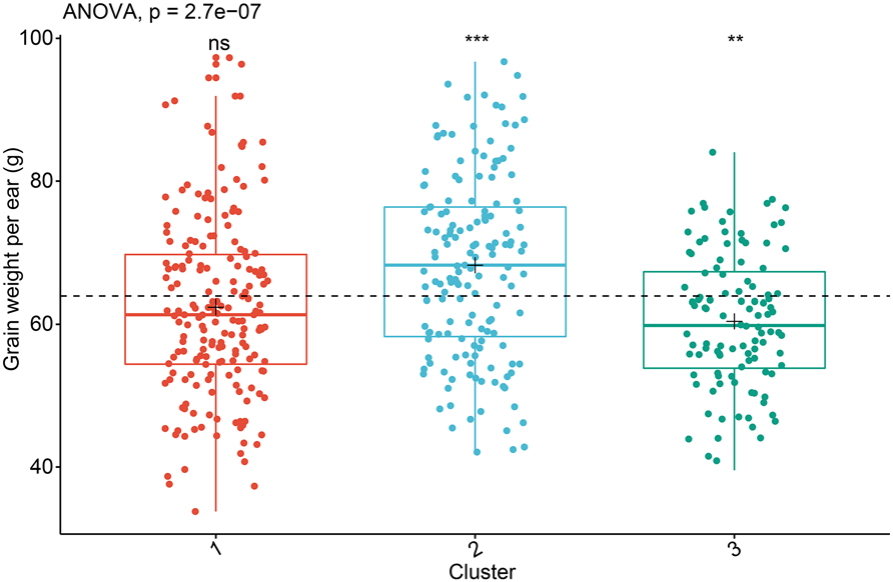
Differences in grain weight per ear between the three genotype clusters. The dashed black line indicated the mean of a phenotypic component from all samples without clustering. The black plus sign indicated the mean of a given phenotypic component from each cluster. An ANOVA was used to determine whether differences exist between three-cluster means. The Wilcoxon rank-sum test was used to compare each cluster against all genotypes without clustering. The following convention for symbols indicated statistical significance: *p* > 0.05 (ns); *p* ≤ 0.01 (**); *p* ≤ 0.001 (***). The total sample size remains 453, excluding missing values

Figure 11 shows the differences in grain weight per ear between the three genotype clusters. The most striking finding to emerge from this analysis is that cluster 3 has a lower grain weight per ear, despite previous analysis showing that it has higher phenotypic components. This finding seems to indicate that lower grain yields occur in genotype samples with higher plant height and higher biomass. Is this true? This result may be explained by the fact that the vast majority of genotypic samples (85.2%) in cluster 3 are TST subpopulations planted in the temperate zone, where the grain yield of such TST populations was low due to the lack of sufficient accumulated temperature during the reproductive-growth stage. Grain yield is a polygenic trait controlled by several genes [62]; phenotypic components do not directly affect the yield but assist in the identification of the genotypes.

Phenotypes of a plant are the expression in observable traits of potential genes comprising the genotype and are determined by its genetic composition, the environment in which it grows, and the interactions between genotype and environment [63, 64]. It is thus possible to measure phenotypic relationships between different genotypes based on available traits [28]. Some phenotypic data extracted from remote sensing images, such as BIOVP and NDVI, clearly differ from the agronomic definition, or “trait.” To distinguish between them, we refer herein to these phenotypic data as phenotypic components. The analysis of phenotypic similarity provides a new inspiration that removes some highly relevant phenotypic components and thereby reduces data redundancy. In other words, the workload can be reduced by making a preliminarily selection of representative phenotypic components as the research objects in the trial design.

### Limitations and Implications of Study

**Fig. 12:**
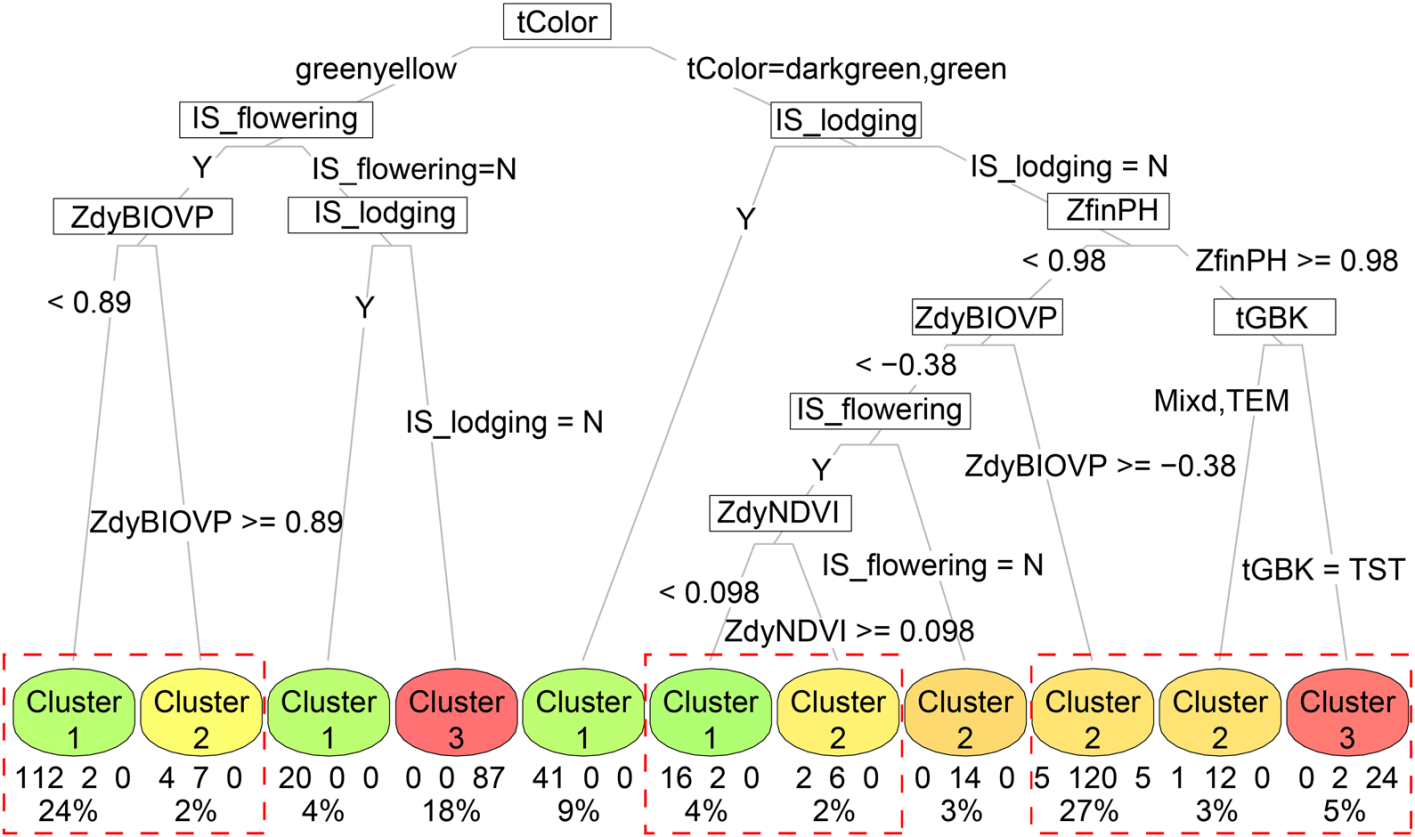
Clustering resolved into decision tree. Each node displayed the predicted class, the number, and the percent of samples. The red-dashed-line rectangle showed a situation where clustering boundaries were blurred

According to the literature, SOM does not really cluster but instead produces a reduced representation of the original data set [46]. Because SOM lacks hierarchical structure, it is impossible to detect higher-order relationships between clusters. In this study, we demonstrate a two-step clustering method that combines hierarchical clustering and self-organizing maps to cluster, analyze, and visualize the mixed continuous and categorical data, providing a very efficient tool for exploratory analysis of genotype co-expression and phenotypic similarity. One problem with self-organizing maps is the occurrence of dead neurons, whose randomly initialized weight vectors farther from any data point prevent them from ever being chosen as BMU, which degrades the learning efficiency. Therefore, if the percentage of dead neurons is large, caution must be applied and the grid size of SOM should be readjusted. After resolving the clusters derived from the SOM map reasonably to a set of clustering rules, approximately 95.2% of the samples can be accurately predicted by using a decision tree. However, the clustering boundaries are not 100% sharp, and some ambiguity remains regarding where on the map a specific neuron (sample) migrates (Fig. 12).

The challenge for plant breeders is to identify and select the plants with the target phenotype controlled by the corresponding genotypes, rather than plants with the target phenotype controlled by environmental impact [65]. For field breeding trials, an effective use of phenotypic data remains challenging, because the environment changes daily throughout the data-collection period [66]. To minimize the impact of the environment on this study, comparatively ideal environmental conditions were attained by doing single-factor experiments and using uniform field-management practices. Even so, the explanation for these results may be the lack of adequate verification, because these phenotypic components were acquired and analyzed only across a single growing season. Further research should be undertaken to investigate the population and provide new multi-year insights into identifying genotypes and their co-expressed patterns and selecting phenotypic similarity.

The capacity of UAV-based HTP to collect and analyze from a few to thousands of breeding plots allows breeders to effortlessly obtain a large number of phenotypic data, and then to efficiently quantify genotypic differences in crop-yield potential, stress resistance, and quality. In this study, the lack of phenotypic data at the late ripening stage prevented the formation of a phenotypic data chain throughout growing season. A hyperspectral sensor is a powerful tool for detecting biological and abiotic stresses, and more crop resistance information can be obtained when it is loaded on the UAV platform. The platform will also be improved to increase payload capacity so that it may be equipped with UAV laser scanning for easy access to plant type and ear-high traits. The phenotypic map and similarity will also more comprehensive when more data from UAV laser scanning and hyperspectral sensor are available. Rich phenotypic data definitely assist breeders with identifying and selecting the best candidate genotypes.

## Conclusions

In this study, we collected for a maize breeding program a short time series of remote-sensing images, including digital and multispectral images, by using an UAV-based HTP platform. The images were used to acquire nine phenotypic components. Here, we propose a framework for pre-screening genotypes and phenotypes based on HTP phenotypic components. The core procedure of this framework can be summarized as follows: we use two-step clustering to identify co-expressed patterns, and then pre-select genotypes. We then use correlation analysis to analyze phenotypic similarity, and then pre-select phenotypic components. This framework gives breeders additional information to quickly identify and select plants that have genotypes that confer desirable phenotypic components from thousands of field plots. The present study also demonstrates that remote sensing is a powerful tool that provides an opportunity to acquire abundant phenotypic components. By using these rich phenotypic components, breeders should be able to more effectively identify and select superior genotypes.

## Additional file

**Additional file 1**. Twenty indices for determining the best number of clusters.

## Data Availability

The datasets analysed during the current study are available from the corresponding author on reasonable request.

## Abbreviations

AGRPH: average growth rate of plant height
AHC: agglomerative hierarchical clustering
ANOVA: analysis of variance
AOI: area of interest
BIOVP: a volume metric used to estimate crop biomass within a plot
Cos2: square cosine and squared coordinates
CSM: crop surface model
DAS: days after sowing
DSM: digital surface model
DEM: digital elevation model
DSMs: digital surface models
HTP: high-throughput phenotyping
NGRDI: normalized green–red difference index
NDVI: normalized difference vegetation index
PCA: principle components analysis
SOM: self-organizing map
UAV: unmanned aerial vehicle
VIs: vegetation indices
