# Supplementary material for: Combining self-organizing maps and biplot analysis to preselect maize phenotypic components based on UAV high-throughput phenotyping platform
Source: Plant Methods. 2019 May 28;15:57. doi: 10.1186/s13007-019-0444-6 (PMC6537385; doi:10.1186/s13007-019-0444-6)
Supplement: Supplementary file 1 — Additional file 1. Twenty indices for determining the best number of clusters. [file 13007_2019_444_MOESM1_ESM.docx]

**Additional file 1: Twenty indices for determining the best number of clusters**

|  | Indexes | Best_nc | Criterion | Reference |
| --- | --- | --- | --- | --- |
| 1 | kl | **3** | Maximum value of the index | [1] |
| 2 | ch | 15 | Maximum value of the index | [2] |
| 3 | hartigan | **3** | Maximum difference between hierarchy levels of the index | [3] |
| 4 | cindex | 2 | Minimum value of the index | [4] |
| 5 | db | **3** | Minimum value of the index | [5] |
| 6 | silhouette | **3** | Maximum value of the index | [6] |
| 7 | duda | 2 | Smallest number of clusters such that index > criticalValue | [7] |
| 8 | pseudot2 | 2 | Smallest number of clusters such that index < criticalValue | [7] |
| 9 | beale | 2 | Number of clusters such that critical value >= alpha | [8] |
| 10 | ratkowsky | **3** | Maximum value of the index | [9] |
| 11 | ball | **3** | Maximum difference between hierarchy levels of the index | [10] |
| 12 | ptbiserial | **3** | Maximum value of the index | [11,12] |
| 13 | frey | 1 | Cluster level before index value < 1.00 | [13] |
| 14 | mcclain | **3** | Minimum value of the index | [14] |
| 15 | gamma | **3** | Maximum value of the index | [15] |
| 16 | gplus | **3** | Minimum value of the index | [16] |
| 17 | tau | 15 | Maximum value of the index | [16] |
| 18 | dunn | **3** | Maximum value of the index | [17] |
| 19 | sdindex | 5 | Minimum value of the index | [18] |
| 20 | sdbw | 15 | Minimum value of the index | [19] |

[1]Krzanowski WJ, Lai YT. A Criterion for Determining the Number of Groups in a Data Set Using Sum-of-Squares Clustering. Biometrics.1988; 44:23–34.

[2]Calinski T, Harabasz J. A Dendrite Method for Cluster Analysis. Communications in Statistics – Theory and Methods.1974; 3:1–27.

[3]Hartigan JA. Clustering Algorithms. New York: John Wiley & Sons; 1975.

[4]Hubert LJ, Levin JR. A General Statistical Framework for Assessing Categorical Clustering in Free Recall. Psychological Bulletin.1976; 83:1072–1080.

[5]Davies DL, Bouldin DW. A Cluster Separation Measure. IEEE Transactions on Pattern Analysis and Machine Intelligence.1979; 1:224–227.

[6]Rousseeuw P. Silhouettes: A Graphical Aid to the Interpretation and Validation of Cluster Analysis. Journal of Computational and Applied Mathematics.1987; 20:53–65.

[7]Duda RO, Hart PE. Pattern Classification and Scene Analysis. New York: John Wiley & Sons; 1973.

[8]Beale EML. Euclidean Cluster Analysis. London: Scientific Control Systems; 1969.

[9]Ratkowsky DA, Lance GN. A Criterion for Determining the Number of Groups in a Classification. Australian Computer Journal.1978; 10:115–117.

[10]Ball GH, Hall DJ. ISODATA: A Novel Method of Data Analysis and Pattern Classi-fication. Menlo Park: Stanford Research Institute; 1965.

[11]Milligan GW. An Examination of the Effect of Six Types of Error Perturbation on Fifteen Clustering Algorithms. Psychometrika.1980; 45:325–342.

[12]Milligan GW (1981). A Monte Carlo Study of Thirty Internal Criterion Measures for Cluster Analysis. Psychometrika.1981; 46:187–199.

[13]Frey T, Van Groenewoud H. A Cluster Analysis of the D-Squared Matrix of White Spruce Stands in Saskatchewan Based on the Maximum-Minimum Principle. Journal of Ecology.1972; 60:873–886.

[14]McClain JO, Rao VR. CLUSTISZ: A Program to Test for The Quality of Clustering of a Set of Objects. Journal of Marketing Research.1975; 12:456–460.

[15]Baker FB, Hubert LJ. Measuring the Power of Hierarchical Cluster Analysis. Journal of the American Statistical Association.1975; 70:31–38.

[16]Rohlf FJ. Methods of Comparing Classifications. Annual Review of Ecology and Systematics.1974; 5:101–113.

[17]Dunn J. Well Separated Clusters and Optimal Fuzzy Partitions. Journal Cybernetics.1974; 4:95–104.

[18]Halkidi M, Vazirgiannis M, Batistakis Y: Quality Scheme Assessment in the Clustering Process. In Principles of Data Mining and Knowledge Discovery; Lyon, France; 2000. p. 265-276.

[19]Halkidi M, Vazirgiannis M. Clustering Validity Assessment: Finding the Optimal Partitioning of a Data Set. In: ICDM’01 Proceedings of the 2001 IEEE International Conference on Data Mining, California; 2001. p. 187–194.
